# Laugier-Hunziker Syndrome: An Uncommon Cause of Oral Pigmentation and a Review of the Literature

**DOI:** 10.1155/2010/525404

**Published:** 2010-07-07

**Authors:** Lucio Montebugnoli, Ivana Grelli, Fabio Cervellati, Cosimo Misciali, Beatrice Raone

**Affiliations:** ^1^Department of Oral Science, University of Bologna, Via San Vitale 59, 40125 Bologna, Italy; ^2^Division of Dermatology, Department of Internal Medicine, Geriatrics, Nephrology, University of Bologna, 401138 Bologna, Italy

## Abstract

Laugier-Hunziker syndrome is a rare benign condition characterized by diffuse oral hyperpigmentation associated with pigmentation of the nails. The syndrome must be included in the differential diagnosis of diffuse oral pigmentation to exclude other conditions with systemic implications. We describe a 43-year-old white woman with the clinical and histological features of Laugier-Hunziker syndrome associated with toenail pigmentation. The correct clinical identification avoids the need for detailed investigations and treatment. We also review the potential causes of oral pigmentation.

## 1. Introduction

Pigmentation is frequently encountered in the oral mucosa. Focal lesions usually need an in-depth examination to exclude a melanoma, while diffuse lesions often have no specific histological features and do not generate prognostic perplexity. However, diagnosis of these lesions is important because they could be a sign of diseases with systemic implications such as Peutz-Jeghers syndrome or adrenal insufficiency [1].

Laugier-Hunziker syndrome is a rare acquired macular hyperpigmentation of oral mucosa and lips frequently associated with longitudinal pigmentation of the nails [2]. The pathogenesis is unknown, but no systemic involvement or malignant predisposition has been described, so the correct clinical identification avoids the need for detailed and potentially hazardous investigations and treatment [3]. Few cases have been reported, and almost all have been published in the dermatological literature [4, 5]. Here we describe a patient with diffuse oral pigmentation associated with a toenail longitudinal pigmented band in which the diagnosis of Laugier-Hunziker pigmentation (Laugier-Hunziker syndrome) was made on the basis of clinical and histological findings.

## 2. Case Report

A 43-year-old white woman was referred to the Department of Oral Sciences of University of Bologna for the evaluation of pigmented oral and labial lesions.

**Figure 1:**
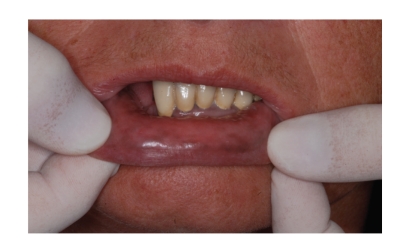
Diffuse macular brown pigmentation on the vermilion of the lower lip.

**Figure 2:**
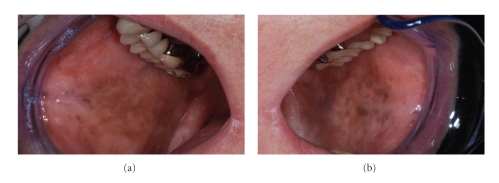
Diffuse macular brown pigmentation on the oral mucosa of right (a) and left (b) cheeks.

**Figure 3:**
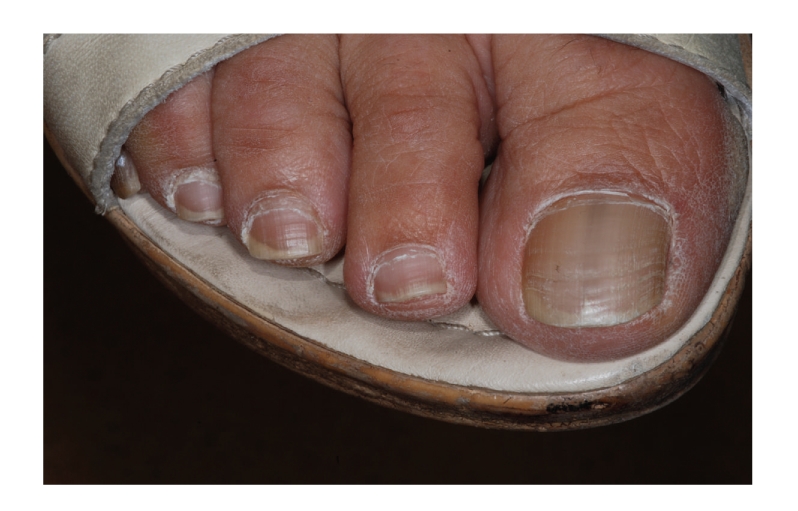
Longitudinal pigmented band on the right great toenail.

The patient had noted such pigmentation but was unable to pinpoint the period of onset. Oral mucosa was completely symptom free and the patient was in a good health; she did not use drugs regularly and did not smoke. There was no family history of abnormal pigmentations of the skin or oral mucosa. Clinical examination disclosed diffuse macular brown pigmentation on the vermilion of the lower lip and oral mucosa on both cheeks (Figures 1 and 2). There was no pigmentation on the gingiva or tongue. The patient referred to longitudinal pigmentation on the fingernails, but it was not visible at clinical examination because of nail varnish. Instead, a single longitudinal pigmented band measuring 3 mm was present on the right first toenail (Figure 3).

**Figure 4:**
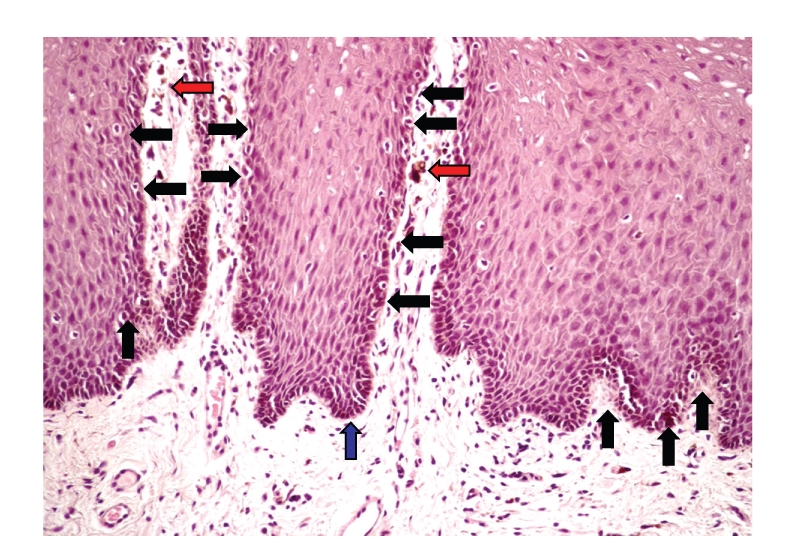
Basal layer pigmentation with increased number of normal melanocytes mostly at chorion epithelial junction in the epithelium (black arrows). Sparse melanophages were noted in the superficial chorion (red arrows), HE X 200.

A biopsy was taken from the oral mucous membrane. The tissue was prepared for routine paraffin processing and hematoxylin and eosin staining. Histology showed increased basal layer pigmentation with an increased number of normal melanocytes mostly at the chorion epithelial junction; sparse melanophages were noted in the superficial chorion (Figure 4). To rule out Addison's disease, the serum, urine cortisol, and ACTH levels were requested and all were within normal levels. Renal and hepatic functions were also normal. Other causes of pigmentation such as drugs or metals were excluded. Upper gastrointestinal endoscopy and colonoscopy were considered but not done as the patient did not present any symptom or clinical sign of intestinal disease and the patient's age and the absence of family history were deemed in contrast with the possibility of Peutz-Jeghers syndrome.

A final diagnosis of Laugier-Hunziker syndrome was made on the basis of clinical and histological features and absence of systemic involvement.

## 3. Discussion

The Laugier-Hunziker syndrome is a rare condition initially described in 1970 involving diffuse oral hyperpigmentation usually beginning in the third to fifth decade of life with a female preponderance [2].

The pigmentation consists of slate to dark brown lenticular or linear macules, solitary or confluent, with well-defined or indistinct margins. The lesions are located most often on the buccal mucosa and lips. Nail involvement is present in about half the patients [6, 7] and consists of pigmented longitudinal bands of varying width and intensity in one or more of the fingernails and/or less often toenails [8]. The hyperpigmentation occurs spontaneously and may progress slowly or remain stable. There are no systemic findings or genetic factors associated with the syndrome.

Histologically, the pigmentation is localized to the basal layer of the epithelium and is thought to be due to an accumulation of melanin in the basal keratinocytes; an increased number of melanophages have also been described in the papillary chorion, while there is some controversy as to the behaviour of the melanocytes. Most reports failed to find an increase in melanocytes [3–9], whereas two reports [4–10] described an increased number of intraepidermal melanocytes that may contribute to the mucosal pigmentation. In accordance with these two reports, an increased number of melanocytes were found in our patient. 

No literature reports have described a progression of Laugier-Hunziker lesions to oral cancer, and therefore all cases must be simply followedup without any specific treatment [11]. However a diagnosis of Laugier-Hunziker syndrome must be established to exclude underlying systemic pathologic conditions.

 Addison's disease is an endocrine disorder due to an insufficient production of cortisol and aldosterone that can present with diffuse hyperpigmentation of the skin and mucous membranes. The oral manifestations are primarily due to an increased level of circulating adrenocorticotropic hormone (ACTH) and may be the first sign of the disease, so the exact interpretation of the lesions is mandatory for prompt diagnosis and to institute appropriate therapeutic strategies. No clinical signs of systemic symptoms such as fatigue, weight loss, and cardiovascular or gastrointestinal disorders were found in our patient, and her plasma levels of cortisol and ACTH were normal [12]. 

Albright's syndrome is genetic disorder of bones, skin pigmentation, and hormonal problems with premature sexual development. Pigmentary changes are not pathognomonic but may include irregular often unilateral truncal pigmentation (café-au-lait macules), macular lip, and genital pigmentation. No nail pigmentation has been reported. The syndrome manifests in childhood and this excludes the possibility of such pathology in our patient.

Diffuse oral pigmentation may also be associated with systemic intake of drugs such as tetracyclines, antimalarials, amiodarone, chemotherapeutic agents, oral contraceptives, phenothiazines, azidothymidine, and ketoconazole. A correct diagnosis will resolve the drug-induced oral mucosal pigmentation following the suspension of the causative drug [13]. No drugs were being taken by our patient.

Smoking may produce oral pigmentation, although it is usually confined to the anterior attached gingival and not associated with pigmentation in other parts of the body [14]. Again, our patient had never smoked.

Inflammatory disorders of the oral mucosa, such as lichen planus, may sometimes be associated with oral pigmentation. In these conditions the oral pigmentation is usually detected together with the specific oral signs of the underlying disease [15]. The results from the oral specimen of our patient did not show any histological characteristic of lichen planus.

The Peutz-Jeghers syndrome (PJS) shares most clinical features with LHS and must be ruled out in case of diffused oral pigmentation because it may be associated with an increased incidence of gastrointestinal as well as genital and mammary tumors. PJS is an autosomal dominant inherited disease with a high degree of penetrance characterized by intestinal polyposis and melanotic macules, particularly of the face and mouth [16]. 

The differential diagnosis between PJS and LHS may be hampered by overlapping clinical and histological features. However, some characteristics may help to differentiate the two syndromes: the appearance of the lesions in infancy or early childhood and the presence of family hyperpigmentation or intestinal polyposis, or pigmentation also on the face, hands, and feet, suggest PJS, while LHS can be assumed when both oral and nail pigmentations are present [17]. 

Our patient presented all the hallmarks of LHS, including middle-age at onset, no family history of the disease or intestinal polyposis, and both oral and toenail pigmentations without local (smoking, oral diseases, etc.) or systemic (drugs, Addison's disease, etc.) causes that could interfere with the diagnosis. 

In conclusion, our patient presented a rare syndrome probably not well known among general dentists, although Mignogna et al. [18] hypothesized a much wider distribution of the disorder than our case would indicate. Dentists should therefore be familiar with the Laugier-Hunziker syndrome as a benign condition not requiring treatment. When a patient presents with diffuse oral pigmentation, detailed history taking and thorough clinical examination including fingernails will establish the diagnosis and exclude local or systemic diseases requiring medical management.
